# Hepatocyte Growth Factor (HGF) Inhibits Collagen I and IV Synthesis in Hepatic Stellate Cells by miRNA-29 Induction

**DOI:** 10.1371/journal.pone.0024568

**Published:** 2011-09-09

**Authors:** Monika Kwiecinski, Andrea Noetel, Natalia Elfimova, Jonel Trebicka, Stephanie Schievenbusch, Ingo Strack, Levente Molnar, Melanie von Brandenstein, Ulrich Töx, Roswitha Nischt, Oliver Coutelle, Hans Peter Dienes, Margarete Odenthal

**Affiliations:** 1 Institute for Pathology, University Hospital Cologne, Cologne, Germany; 2 Department of Internal Medicine I, University of Bonn, Bonn, Germany; 3 Department of Gastroenterology and Hepatology, University Hospital of Cologne, Cologne, Germany; 4 Department of Dermatology, University Hospital of Cologne, Cologne, Germany; 5 Department of Internal Medicine, University Hospital Cologne, Cologne, Germany; University of Bristol, United Kingdom

## Abstract

**Background:**

In chronic liver disease, hepatic stellate cells (HSC) transdifferentiate into myofibroblasts, promoting extracellular matrix (ECM) synthesis and deposition. Stimulation of HSC by transforming growth factor-β (TGF-β) is a crucial event in liver fibrogenesis due to its impact on myofibroblastic transition and ECM induction. In contrast, hepatocyte growth factor (HGF), exerts antifibrotic activities. Recently, miR-29 has been reported to be involved in ECM synthesis. We therefore studied the influence of HGF and TGF-β on the miR-29 collagen axis in HSC.

**Methodology:**

HSC, isolated from rats, were characterized for HGF and Met receptor expression by Real-Time PCR and Western blotting during culture induced myofibroblastic transition. Then, the levels of TGF-β, HGF, collagen-I and -IV mRNA, in addition to miR-29a and miR-29b were determined after HGF and TGF-β stimulation of HSC or after experimental fibrosis induced by bile-duct obstruction in rats. The interaction of miR-29 with 3′-untranslated mRNA regions (UTR) was analyzed by reporter assays. The repressive effect of miR-29 on collagen synthesis was studied in HSC treated with miR-29-mimicks by Real-Time PCR and immunoblotting.

**Principal Findings:**

The 3′-UTR of the collagen-1 and −4 subtypes were identified to bind miR-29. Hence, miR-29a/b overexpression in HSC resulted in a marked reduction of collagen-I and -IV synthesis. Conversely, a decrease in miR-29 levels is observed during collagen accumulation upon experimental fibrosis, in vivo, and after TGF-β stimulation of HSC, in vitro. Finally, we show that during myofibroblastic transition and TGF-β exposure the HGF-receptor, Met, is upregulated in HSC. Thus, whereas TGF-β stimulation leads to a reduction in miR-29 expression and de-repression of collagen synthesis, stimulation with HGF was definitely associated with highly elevated miR-29 levels and markedly repressed collagen-I and -IV synthesis.

**Conclusions:**

Upregulation of miRNA-29 by HGF and downregulation by TGF-β take part in the anti- or profibrogenic response of HSC, respectively.

## Introduction

Progressive liver fibrosis due to chronic viral hepatitis, autoimmune, metabolic or hereditary disorders is a leading cause of morbidity and mortality in the Western world (reviewed in [1], [2], [3]). Regardless of the underlying etiology, liver fibrosis is characterized by an excessive deposition and reorganization of extracellular matrix (ECM) with a dramatic increase in non-collagenous and collagenous ECM proteins. The fibrillar collagen type I, is encoded by two different genes, col1A1 and col1A2, and accounts for 36% of the total collagens in ECM of healthy liver. During liver fibrogenesis, collagen type I is the predominant isoform deposited into the perisinusoidal space. However, collagen type IV, that constitutes less than 10% of total collagen in the normal liver, is most dramatically upregulated in fibrosis [4], [5], [6].

In the fibrotic liver, hepatic stellate cells (HSC) undergo myofibroblastic transdifferentiation. These myofibroblastic HSC are regarded as the main source of ECM production [1], [3], [7], [8] although portal myofibroblasts, infiltrating fibroblasts and fibrocytes may also participate in the synthesis and restructuring of the connective tissue [9], [10]. HSC get activated in response to chronic liver injury by proinflammatory and profibrogenic mediators such as transforming growth factor-β (TGF-β) [11], [12] and platelet-derived growth factor β [13], [14]. TGF-β is recognized as the main profibrogenic mediator, triggering the myofibroblastic transition of HSC. Furthermore, it promotes the synthesis of ECM proteins, and inhibits expression and activity of matrix degrading enzymes in HSC [15].

TGF-β stimulated matrix production and deposition has been shown in a wide range of models of experimental fibrosis [16], [17] and in patients with chronic hepatitis and cirrhosis [18], [19], [20]. Interestingly, there is good evidence for hepatic growth factor (HGF) opposing TGF-β signalling by reducing TGF-β mRNA levels [21]. HGF is a multifunctional cytokine that elicits mitogenic, motogenic, and morphogenic properties [22], [23] by activation of the tyrosine kinase receptor Met, a product of the proto-oncogene *c-met* [24], [25]. In addition, HGF is known to inhibit accumulation of extracellular matrix and development of hepatic fibrosis *in vivo* [26], [27], [28]. TGF-β can in turn dramatically suppress HGF mRNA expression in HSC, demonstrating the reciprocal effects of these cytokines on ECM accumulation [29].

The synthesis of extracellular matrix proteins is modulated by microRNA-29 (miR-29) in extrahepatic tissue [30], [31], [32], [33]. Recent reports suggest that miR-29 is also involved in the synthesis of collagen type I in liver fibrosis [34], [35]. The miR-29 family consists of miR-29a, miR-29b (b_1_, b_2_), and miR-29c, which differ in only two or three nucleotides, respectively. The genes for miR-29a and miR-29b_1_ are both located on chromosome 7, whereas the genes for miR-29c and miR-29b_2_ are located on chromosome 1. Each gene pair is transcribed in tandem resulting in a common pri-miRNA from which the mature miR-29 members are released after further processing [36], [37].

In the present study, we investigate the role of the members of the miR-29 family in HGF mediated repression of collagen synthesis. We demonstrate that miR-29 is not only involved in collagen type I but also in type IV synthesis of myofibroblastic HSC. The importance of miR-29 in hepatic collagen homeostasis is underlined by our *in vivo* data that shows the lack of miR-29 in severe experimental fibrosis after bile duct obstruction. This loss of miR-29 is suggested to be due to the response of HSC to exposure to profibrogenic mediators as shown by our *in vitro* findings on TGF-β stimulated HSC. Whereas TGF-β stimulation leads to decreased miR-29 levels, but to pronounced upregulation of collagen synthesis, HGF stimulation leads to elevated miR-29 expression, but to repression of collagen synthesis. Thus, our data provide detailed evidence for the antifibrotic action of miR-29 in response to HGF signalling that is counteracted by the profibrotic growth factor TGF-β.

## Materials and Methods

### Isolation of primary HSC from rat, cell culture and stimulation of HSC

Isolation and plastic-induced myofibroblastic *in vitro* activation of primary HSC was performed as previously described [38], [39]. Cells were maintained in Dulbecco's modified Eagles medium (DMEM) with 10% fetal calf serum (FCS) at 37°C and 5% CO_2_ in a humidified atmosphere. HSC at day 3 are considered as quiescent relative to day 7 of HSC culture, when they express all features of myofibroblasts and considered as myofibroblastic cells. For miR-29 transfection assays, HSC-T6 cells (kindly provided by SL Friedman) were cultured in agreement to the previous description [40], [41]. In order to stimulate HSC with TGF-β cells were cultured to 90% confluency in DMEM containing 10% FCS and starved out for 24 h in DMEM containing 0.5% FCS. For HGF stimulation HSC-T6 cells were cultured to a confluency of 70% in DMEM containing 10% FCS. After 24 h 10 ng/ml TGF- β1 (R&D Systems, Wiesbaden-Nordenstadt, Germany), or 40 ng/ml recombinant hHGF (Dianova, Hamburg, Germany) was added and cells were cultured for a further 24 h.

### Bioinformatics

In order to search miRNA targets, the databases miRanda (http://www.microrna.org) [42], [43], Targetscan (http://www.targetscan.org) [44], and Pictar (http://pictar.bio.nyu.edu) [45] were queried.

### Plasmid constructs

**Figure 1:**
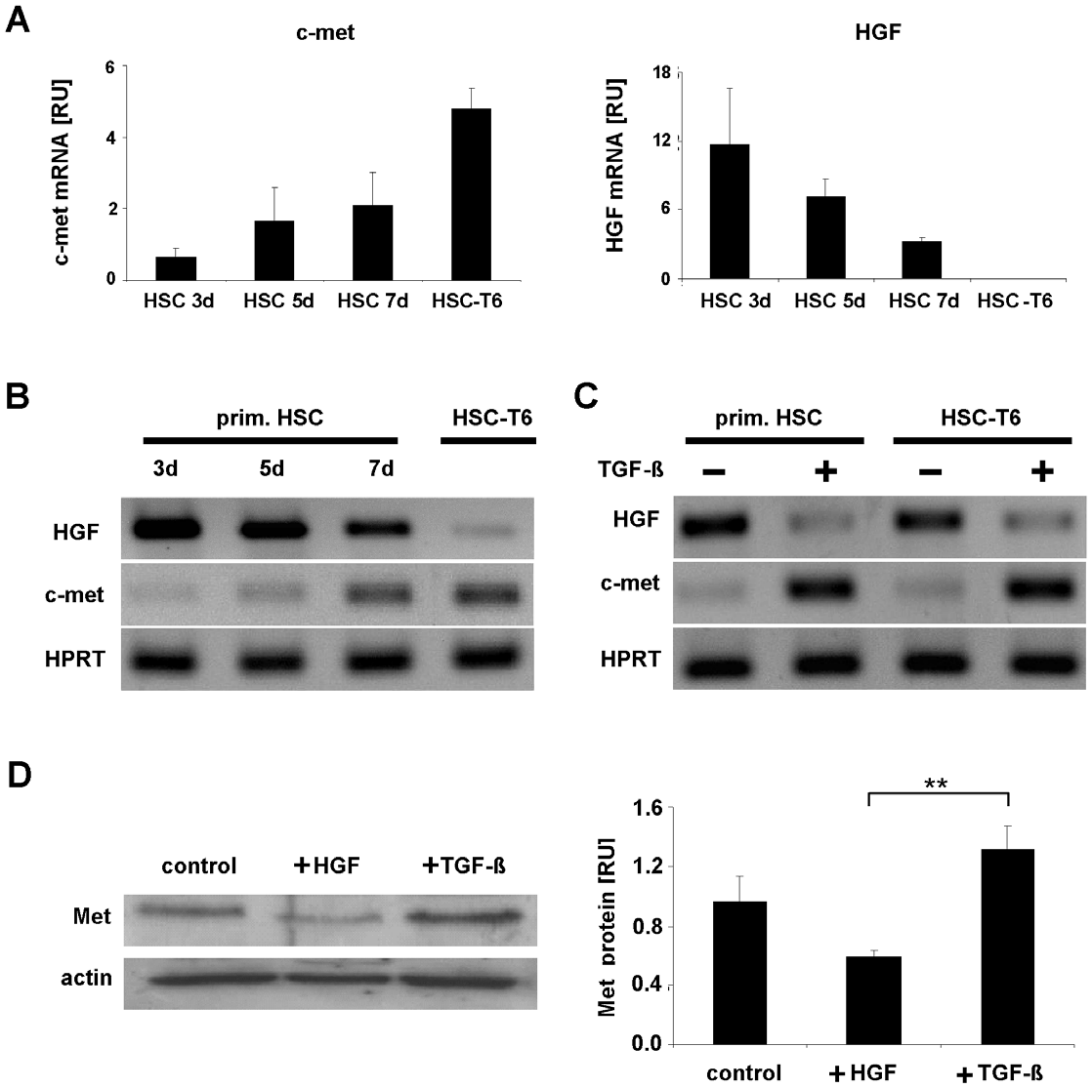
Met and HGF expression in primary and immortalized HSC. Total RNA was extracted from primary rat HSC after the third, fifth and seventh day of cell culture (3d, 5d, 7d) and from myofibroblastic HSC-T6 cells. Subsequently, c-met and HGF expression was determined (A–C). The c-met and HGF transcript levels were quantified by Real-Time PCR and normalized using HPRT as house-keeping gene (A–C). The expression of c-met increased during the differentiation process of primary HSC and achieved the highest level in the immortalized cell line HSC-T6 representing a myofibroblastic phenotype [41], whereas HGF levels were shown to be opposite (A, B). Primary HSC at the 3rd culture day (prim. HSC) and immortalized HSC (HSC-T6) were either untreated (−) or stimulated with TGF-β (+) and RNA levels of c-met and HGF were analyzed by Real-Time PCR (C). The Met protein expression in HGF and TGF-β stimulated HSC-T6 (+HGF, +TGF-β) in comparison to untreated HSC-T6 cells (control) was shown by Western blot analysis (D).

The 3′-UTR of collagen 4A1 (col4A1) and collagen 4A5 (col4A5) transcripts bearing putative miRNA-binding sites for miR-29 were amplified from genomic rat DNA using the primer shown in the Table S1. Furthermore, the putative binding sites of collagen 1A1 (col1A1), collagen 1A2 (col1A2), collagen 4A1(col4A1), and collagen 4A5 (col4A5) as well as the corresponding mutants containing two point mutations were cloned by insertion of the dimerized oligonucleotides shown in the Table S2. Amplicons and oligonucleotide dimers were cloned downstream of the renilla luciferase reporter of the psiCHECK^TM^-2 vector (Ambion, Austin, USA) (Figure 1 A).

### miRNA transfection and reporter assays

miRNA mimicking miR-29a, miR-29b and a scrambled miRNA control were obtained from Dharmacon (Lafayette, USA). HSC were seeded in 6-well plates, incubated overnight to a density of 90% and transfected using Lipofectamine 2000 (Invitrogen, Karlsruhe, Germany) as instructed. Afterwards, the cells were lysed either in SDS-lysis buffer (15 mM Tris-HCL pH 6.8, 0.5% SDS, 2.5% glycerol) containing protease inhibitors (Roche Applied Science, Mannheim, Germany) or in Qiazol (Qiagen, Hilden, Germany) and stored for subsequent protein or RNA analyses, respectively.

For reporter assays, cells were seeded in 24-well plates to reach 90% confluency overnight. 50 nM miRNA and 2 µg/ml psiCHECK^TM^-2 reporter was used for transfection using Lipofectamine 2000. The level of miR-29 transfected into HSC-T6 cells was analyzed by Real-Time PCR, demonstrating stable levels between 8 h and 48 h post-transfection. Cells were therefore harvested 24 hours after transfection and luciferase activity in the samples was determined using the Dual Luciferase Reporter Assay System (Promega, Mannheim, Germany) according to the manufacturer's recommendations. All samples were measured in triplicate and experiments were each performed three times.

### Total RNA extraction

Total RNA from snap-frozen cell culture and tissue samples was isolated using the Qiazol reagent as instructed (Qiagen, Hilden, Germany). Rat liver tissues were homogenized using the Precellys 24 tissue homogenizer (Peqlab Biotechnologie, Erlangen, Germany) prior to extraction. Total RNA yields were determined by A_260_ measurement using the ND-1000 NanoDrop spectrophotometer (NanoDrop, Wilmington, USA) and RNA integrity was assessed by microcapillary electrophoresis (2100 BioAnalyser, Agilent Technolologies, Waldbronn, Germany).

### Quantifying microRNA by Real-Time PCR

microRNA was quantified by a two-step Real-Time PCR using the miScript-Reverse Transcription Kit and the miRNA-SYBR Green PCR Kit (Qiagen, Hilden, Germany). The first step includes total RNA polyadenylation and reverse transcription, followed by Real-Time PCR. Primers used for cDNA synthesis and Real-Time PCR were selected and purchased from the GeneGlobe Search Center (Qiagen). 2 ng of all samples were used and processed in triplicate in agreement with the supplier's guidelines. Cellular miRNA levels were normalized using RNU6 as reference RNA.

### Transcript quantification by Real-Time PCR

Total RNA concentrations were estimated by A_260_ measurement. 1 µg of total RNA was reverse transcribed using the High Capacity cDNA Reverse Transcription Kit (Applied Biosystems Applera, Darmstadt, Germany) as instructed. 10 ng of cDNA were used for Real-Time PCR using specific primers listed in Table S1 B and Power SYBR Green PCR Mastermix (Applied Biosystems Applera). All reactions were performed in triplicate. Transcript levels were evaluated by absolute quantification using an on-line standard curve and corrected by normalization to the house-keeping gene, hypoxanthine phosphoribosyltransferase (HPRT).

### Immunoblotting

Total protein concentrations were determined using the BCA protein assay (Thermo Scientific, Bonn, Germany). Equal amounts of protein (10 µg) were resolved on 4–12% SDS-polyacrylamid gels (Biorad, München, Germany), electrotransferred to Hybond ECL nitrocellulose membranes (Amersham Biosciences, München, Germany) and immunostained with the anti-collagen I (1∶1000), anti-collagen IV (1∶5000) antibodies, both from Abcam (Cambridge, UK), or with anti-Met antibodies (1∶500) from Santa Cruz (Heidelberg, Germany), or anti-actin antibodies (1∶5000) from Sigma-Aldrich (Hamburg, Germany). Signals were visualized and quantified using enhanced chemiluminescence (ECL) substrate and a FLUORCHEM®FC2 Alpha Ease scanner (Biozym, Hess. Oldendorf, Germany).

### Induction of biliary fibrosis by bile duct occlusion in rats

10 male Wistar rats (250–300 g) were housed in individual cages with free access to regular chow and water. All experiments were conducted in accordance with National Health and Medical Research Committee Guidelines for Animal Experimentation and approved by the local veterinary authority of North Rhine Westphalia (reference number: K07,04/03). 5 animals were sham operated and 5 animals were subjected to bile duct occlusion (BDO) as described previously [46], [47]. Livers were snap frozen in liquid nitrogen and stored at minus 80°C for analysis of mRNA, protein expression, and hydroxyproline content as described before [47]. Liver tissue from liver segment IV was fixed in 4% buffered formaldehyde solution and embedded in paraffin for histopathological evaluation after Gomori, HE and Elastica van Gieson staining.

### Statistical analysis

Statistical analyses were performed using SPSS software 17 (Chicago, IL, USA). Data obtained from rat tissues was shown as boxplots and subjected to analyses of variance (ANOVA) using Dunnetts post-hoc comparison. Bar graphs show means ± SEM and values of *p* < 0.05 were considered statistically significant.

## Results

HGF is known to suppress fibrosis [26], [27], [28] and its antagonistic interaction with the TGF-β pathway leading to collagen suppression has recently been shown in renal fibroblasts [48], [49]. Here, we studied the function of HGF in collagen synthesis of primary HSC undergoing myofibroblastic activation after primary culture for up to 7 days. Myofibroblastic transition of primary HSC in culture resembles the process *in vivo* and is characterized by the loss of vitamine A, increased proliferation, and elevated expression of profibrogenic mediators and various connective tissue proteins [5], [11], [29], [39]. The most prominent feature of the myofibroblastic transdifferentiation process, *in vitro* and *in vivo,* is the enhanced expression of α-smooth muscle actin (SMA). Therefore, we first demonstrated the process of myofibroblastic transdifferentiation by analysis of the induced SMA expression of isolated rat HSC in culture (Figure S1).

The myofibroblastic transition of primary HSC was accompanied by a reduction in HGF transcripts and a simultaneous increase in the HGF receptor, c-met (Figure 1 A, B). The opposite effects in HGF and c-met expression were most pronounced in HSC of the HSC-T6 cell line, representing fully transdifferentiated myofibroblastic HSC [41]. In HSC-T6 HGF transcripts were lost while the c-met levels were high (Figure 1 A). Interestingly, TGF-β stimulation of both, HSC at day 3 of primary culture and of myofibroblastic HSC-T6 cells was capable of downregulating HGF and further upregulating c-met (Figure 1 C). In line with the transcriptional response, Met protein synthesis increased after TGF-β exposure in HSC, but decreased after stimulation with HGF (Figure 1 D).

### HGF generates antifibrotic effects in HSC by repression of the collagen I and collagen IV synthesis and attenuating the TGF-β expression

**Figure 2:**
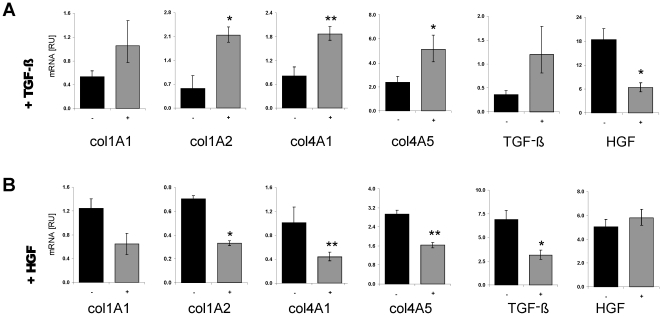
Contrary influence of TGF-β and HGF stimulation on collagen, but also on TGF-β and HGF expression, itself. Collagen 1A1 (col1A1), collagen 1A2 (col1A2), collagen 4A1 (col4A1), and collagen 4A5 (col4A5), HGF and TGF-β mRNA levels were compared by Real-Time PCR in untreated (−) and in HSC-T6 cells stimulated either with TGF- β (+TGF- β) (A) or with HGF (+HGF) (B).

TGF-β is a highly potent profibrogenic growth factor that activates and promotes collagen synthesis in HSC [4], [12]. Whereas stimulation of HSC with TGF-β induced the expression of various collagen subunits such as col1A1, col1A2, col4A1, col4A5 in addition to TGF-β, itself, the HGF transcript levels were markedly repressed (Figure 2 A).

In contrast to TGF-β treatment, the stimulation of HSC with HGF lead to reduced expression of col1A1, col1A2, col4A1, and col4A5, as well as TGF-β expression. However, there was no remarkable change of HGF expression (Figure 2 B).

### miR-29a and miR-29b regulate collagen I and IV synthesis in activated HSC

Since miRNA has been shown to profoundly affect gene regulation, we screened different databases, in order to detect miRNA candidates that inhibit collagen I and IV synthesis in HSC upon HGF stimulation. In the 3′UTR sequences of the collagen-1 and collagen-4 mRNA, potential binding sites for the members of the let-7 family were detected. A number of other miRNA species that might affect collagen type-I and -IV synthesis, such as miR-106a/b, miR-20a/b, miR-26a/b, miR-374a/b, miR-186 were also identified by *in silico* analyses (summarized in Table 1). Strikingly, all of the collagen-1 and -4 transcripts contained binding sites for members of the miRNA-29 family in their 3′UTR (Table 1). Since miR-29 is thought to be involved in modulating expression of extracellular matrix components (Table S3) [30], [31], [32], [34], this makes them promising candidates for collagen I and IV repression after HGF stimulation of HSC.

**Table 1:** miRNAs with putative binding sites in transcripts of collagen 1 and 4.

*Putative Targets*		*col1A1*	*col1A2*	*col4A1*	*col4A2*	*col4A3*	*col4A4*	*col4A5*	*col4A6*
miRNAs with putative bindings sites in collagen 1 and 4 mRNA	hsa-let-7a	+	+	+					+
	hsa-let-7b	+	+	+					+
	hsa-let-7c	+	+	+					+
	hsa-let-7d	+	+	+					+
	hsa-let-7e	+	+	+					+
	hsa-let-7f	+	+	+					+
	hsa-let-7g	+	+	+					+
	hsa-let-7i	+	+	+					+
	hsa-miR-106a			+	+	+		+	
	hsa-miR-106b			+	+	+		+	
	hsa-miR-125a-5p		+	+		+	+		
	hsa-miR-1297		+	+	+	+			
	hsa-miR-17			+	+	+		+	
	hsa-miR-202		+	+		+	+		
	hsa-miR-20a			+	+	+		+	
	hsa-miR-20b			+	+	+		+	
	hsa-miR-26a		+	+	+	+			
	hsa-miR-26b		+	+	+	+			
	hsa-miR-326			+		+	+		+
	hsa-miR-374a			+		+		+	+
	hsa-miR-374b			+		+		+	+
	hsa-miR-410			+			+	+	+
	hsa-miR-544		+	+		+	+		
	hsa-miR-93			+	+	+		+	
	hsa-miR-98	+	+	+					+
	*** more than 4 putative binding sites in the 3′UTR of col1 and col4 subunit***s								
	hsa-miR-23a		+	+	+		+	+	
	hsa-miR-23b		+	+	+		+	+	
	hsa-miR-381	+	+	+		+			+
	hsa-miR-495		+	+		+		+	+
	hsa-miR-300	+	+	+		+			+
	hsa-miR-590-3p		+	+		+	+	+	
	hsa-miR-186	+	+	+	+	+		+	+
	*** putative binding sites in the 3′UTR of all col1 and col4 subunits***								
	hsa-miR-29a	+	+	+	+	+	+	+	+
	hsa-miR-29b	+	+	+	+	+	+	+	+
	hsa-miR-29c	+	+	+	+	+	+	+	+

**Figure 3:**
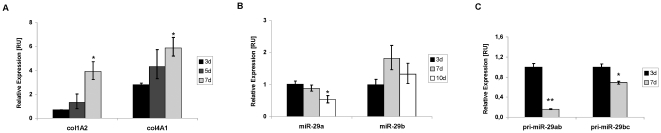
Reciprocal expression of the collagen subunits, col1A1 and col4A2, and primary and mature miR-29. RNA of primary HSC in the quiescent stage (day 3 of primary culture) and after myofibroblastic activation (day 7 of primary culture) was analyzed for mRNA col1A2 and col4A1 levels (A), as wells as for the levels of mature miR-29a and miR-29b (B), and the primary transcripts of the miR-29a/b and miR-29b/c gene (C).

First, we analyzed the synthesis of miR-29 in quiescent and myofibroblastic HSC. Interestingly, the expression of mature miR-29a, but not of miR-29b, was contrary to the collagen expression during myofibroblastic transition. Thus, whereas col1A2 and col4A1 collagen expression was highly increased in myofibroblastic HSC, the levels of miR-29a were suppressed (Figure 3 A, B). Analysis of the transcriptional levels of the primary precursor miRNA (pri-miRNA) revealed that pri-miR-29b/c transcript levels are only moderately suppressed, but that the miR-29a/b gene of chromosome 7 undergoes predominant transcriptional repression upon myofibroblastic transition of HSC.

**Figure 4:**
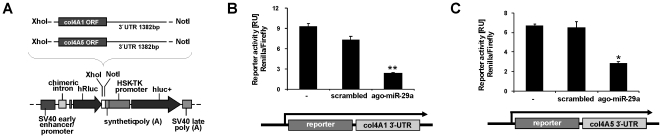
miR-29 interaction with the 3′-UTR of col4A1 and col4A5 transcripts. The 3′-UTR of col4A1 (1382 bp) or col4A5 (786 bp) mRNA was subcloned downstream of the Renilla luciferase reporter (hRluc) of the psiCHECK^TM^-2 vector (A). Reporter plasmids were cotransfected into HSC-T6 in combination with scrambled miRNA or miR-29a mimic (ago-miR-29a), respectively, and luciferase reporter expression was determined by the hRluc luminescence measurement normalized to firefly luminescence (hluc+) (B–C).

In order to study miR-29 function in collagen synthesis, we inserted the 3′-UTR sequences downstream of a luciferase reporter (Figure 4 A). During progression of liver fibrosis, collagen IV is most prominently upregulated among ECM components [6]. Therefore, in addition to collagen-1, the collagen-4 mRNA could be an important target of miR-29 in HSC after HGF stimulation. Indeed, insertion of the 3′-UTR of col4A1 and col4A5 downstream of the luciferase reporter gene lead to a reduction in luciferase expression after treatment of HSC with ago-miR-29a, mimicking miR-29a (Figure 4).

**Figure 5:**
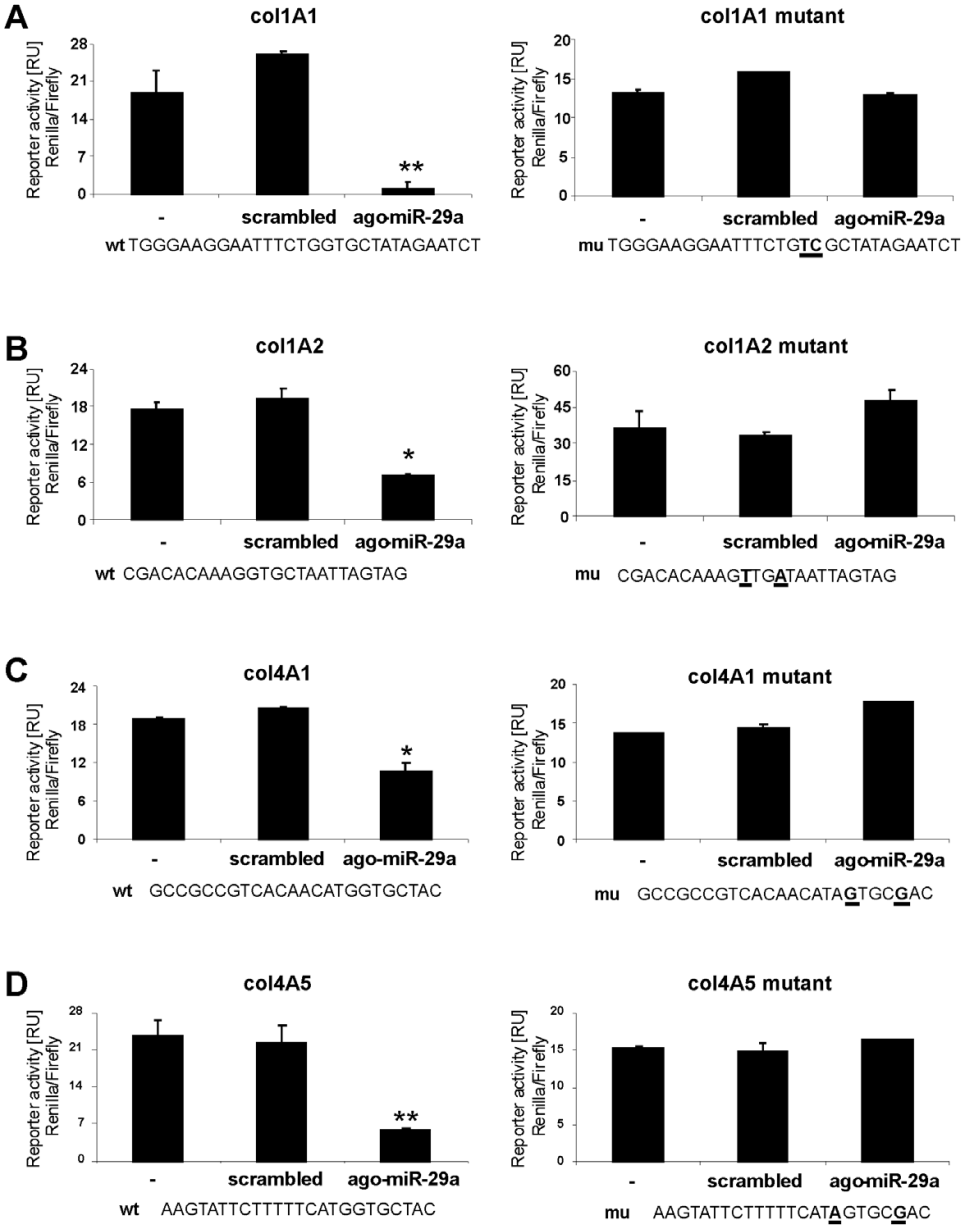
Interaction of miR-29a with the binding regions of col1A1, col1A2, col4A1 and col4A5 3′-UTR transcripts. Putative miR-29 binding sites in the collagen col1A1 (A), col1A2 (B), col4A1 (C) and col4A5 (D) 3′-UTR (wt) and the corresponding mutated sequences (mu) carrying two point mutations (bold and underlined) were cloned into psiCHECK^TM^-2 vector. The reporter plasmids were co-transfected into HSC-T6 cells with either scrambled miRNA or miR-29a mimic (ago-miR-29a), respectively. Insertion of the miR-29 binding sites (wt), but not with mutated binding site (mu), resulted in reduced reporter gene expression by miR-29a treated HSC (A–D).

Among the putative miR-29 binding sites of the collagen mRNA (shown in the Table S4), the following sites were chosen for our further analyses, due to the suggestions of Bartel et al. [50] to function most likely as an inhibitory miR-29 interaction sequence: the region of positon 29–35 in the col4A1 3′-UTR, of postion 404–410 in the col4A5, positon 903–909 in the col1A1, and of position 506–512 in the col1A2 3′-UTR (Table S4). To demonstrate the specificity of miR-29 for the binding sites, in the 3′-UTRs two point mutations were incorporated to abolish the putative miR-29 recognition sequences of the collagen-4 mRNA (col4A1, col4A5) and collagen-1 transcripts (col1A1 and col1A2). Co-transfection of HSC-T6 with the reporter plasmids and ago-miR-29a reduced reporter activity of the wild type controls, but not of the mutated collagen type I and IV constructs (Figure 5 A–D).

**Figure 6:**
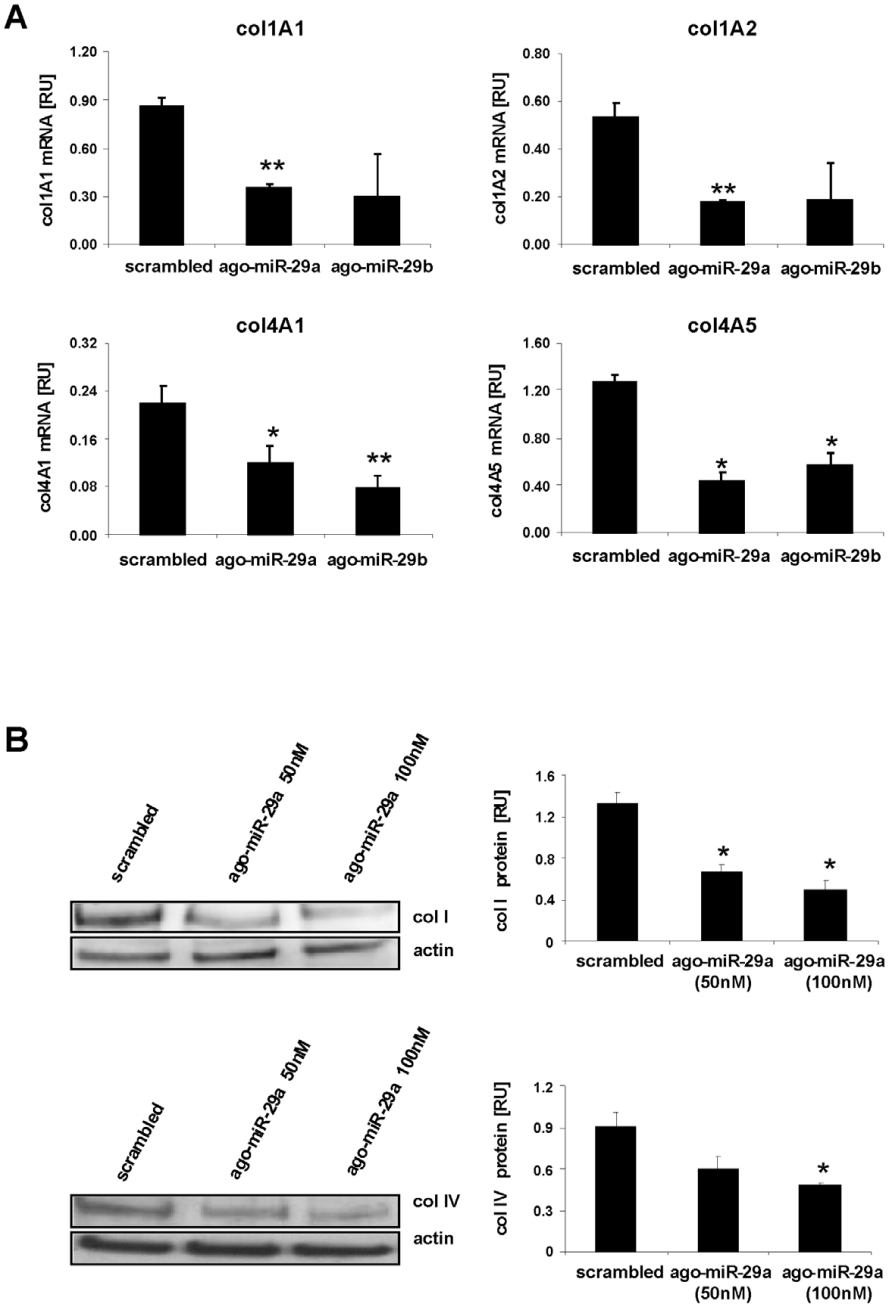
Repression of collagen synthesis by miR-29a and miR-29b. mRNA quantification of collagen subunits in HSC treated either with miR-29a, miR-29b (ago-miR-29a, ago-miR-29b), or scrambled miRNA by Real-Time PCR (A). The col1A1, col1A2, col4A1, and col4A5 mRNA levels were normalized to HPRT expression. Furthermore, collagen I and IV protein levels (col I, col IV) were determined by immunoblotting and subsequent densitometric quantification (B).

Furthermore, transfection of ago-miR-29a and ago-miR-29b into HSC suppressed transcription and protein synthesis of collagen type I and IV ((Figure 6A, B). Together, these data demonstrated that miR-29 specifically inhibits transcription and protein expression of collagen I and IV. In order to analyze if other features of myofibroblastic transition were affected by miR-29, we determined SMA expression in miR-29 treated HSC, because increased SMA assembly is one of the most important features of myofibroblastic transition (Figure S1 A). However, overexpression of miR-29 in myofibroblastic HSC did not affect the expression of SMA (Figure S1 D).

### miR-29a and miR-29b are downregulated during experimental liver fibrosis in rat

**Figure 7:**
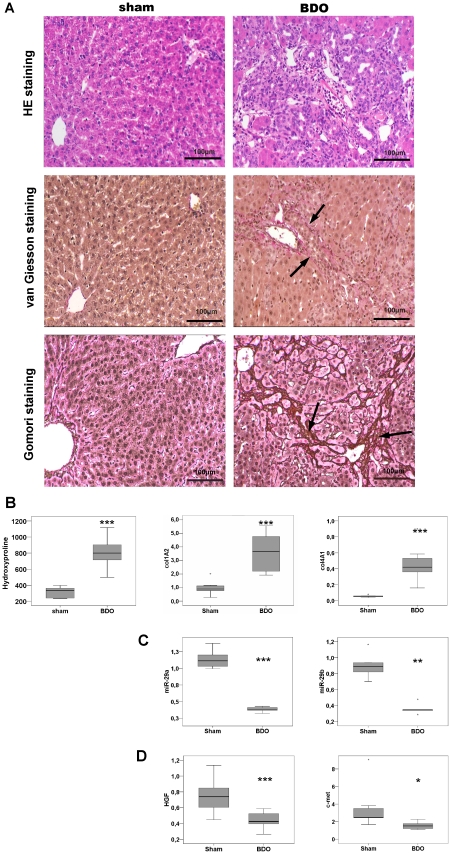
Reduced miR-29a and miR-29b expression in livers after BDO. Liver tissues from bile duct obstructed (BDO) rats showed hepatic inflammation and fibrosis shown by haematoxylin-eosin (HE), van Gieson, and Gomori staining (A). HE histology clearly shows the loss of liver architecture and inflammation after BDO. van Gieson and Gomori stainings revealed the enhancement of connective tissue and the formation of septa (arrows indicate matrix deposition stained in red by van Gieson or in dark brown by Gomori, respectively). Hydroxyproline levels in livers of sham operated (N = 5) and of BDO rats (N = 5) were determined and col1A2 and col4A2 mRNA levels were quantified by Real Time PCR (B). Additionally, miR-29a and miR-29b levels (C) and HGF and c-met transcripts (D) were quantified by Real-Time PCR. While miR-29 and HGF expression was significantly reduced (C,D), hydroxyproline and collagen mRNA were signifcantly increased in BDO livers (B) (*: p<0.05 **:p<0.01; ***: p<0.001).

Next, we induced experimental fibrosis by bile duct occlusion (BDO) in rats and studied the miRNA-29 expression during liver fibrogenesis. After four weeks of BDO both hepatic inflammation and collagen accumulation was evident in histological sections (Figure 7 A). Hydroxyproline determination indicates significant enhancement of matrix deposition in BDO treated livers (Figure 7 B). Thus, BDO induced severe liver fibrosis, when compared to livers of sham-operated animals. Consistent with the antifibrogenic and inhibitory function of miR-29 in collagen I and IV synthesis and the increase of col1A2 and col4A2 (Figure 7 B), we observed a significant reduction in hepatic miR-29a and miR-29b levels in this experimental BDO model (Figure 7 C). This loss of the miR-29a and miR-29b in fibrotic BDO treated livers is attended by reduced levels of HGF upon fibrosis (Figure 7 D).

### miR-29 synthesis in HSC is suppressed by TGF-β, but promoted by HGF

**Figure 8:**
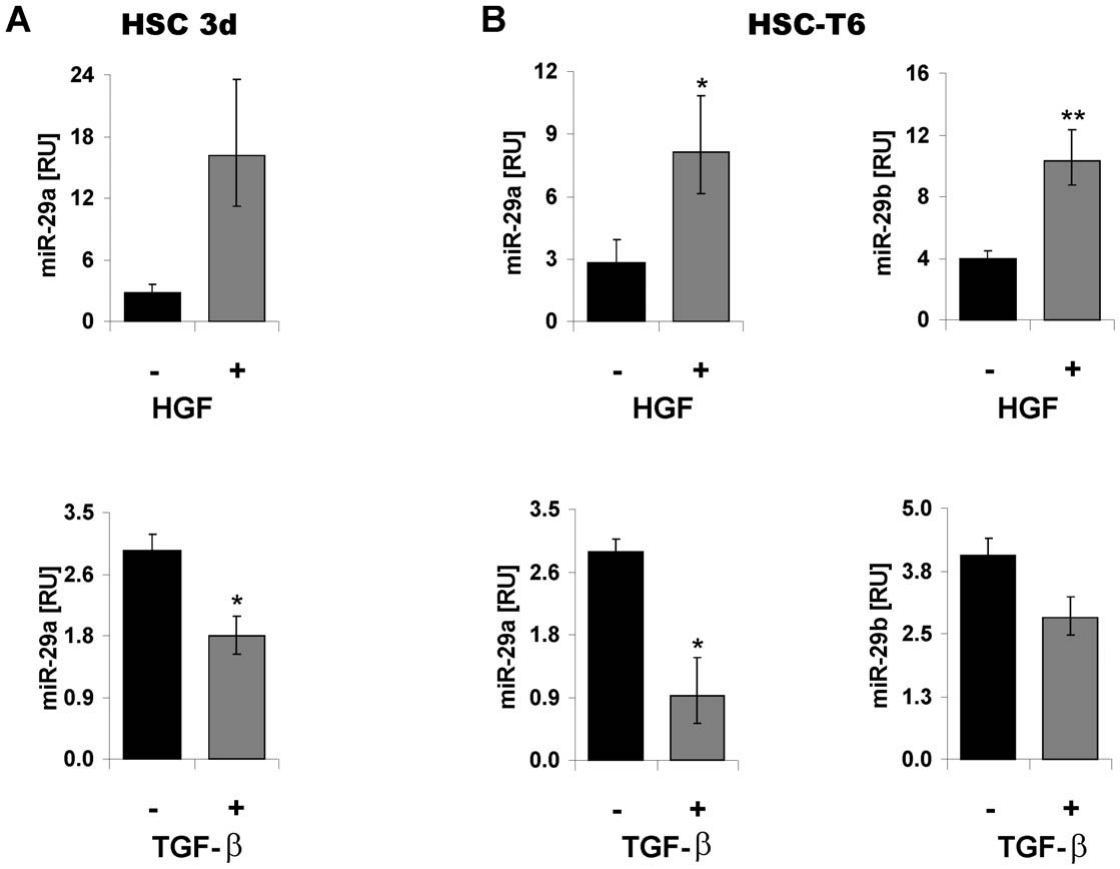
Contrary effects of HGF and TGF-β on miR-29 expression in HSC. miR-29a expression in HSC after 3 days (3d) of primary culture (A) and miR-29a and miR-29b expression in myofibroblastic HSC-T6 (B). HSC were not stimulated (−) or treated (+) with either TGF-β or hHGF and miR-29a/b levels were determined by Real Time-PCR analysis.

Stimulation of primary HSC and myofibroblastic HSC-T6 cells with TGF-β decreased significantly the expression of miR-29a and miR-29b *in vitro* (Figure 8). In contrast, the incubation of primary and transdifferentiated HSC with the recombinant hHGF elicited a marked upregulation of the miR-29a/b levels (Figure 8). To address the question if the antagonistic influence of TGF-β and HGF can be also observed in other species or fibroblastic cells of other organs, we stimulated primary skin fibroblasts with both TGF-β and HGF. In agreement with the data on rat HSC, miR-29 is repressed by TGF-β, but upregulated by HGF (Figure S2).

Therefore, miR-29 expression appears to be reciprocally regulated by profibrogenic (TGF-β) and antifibrogenic growth factors (HGF) in HSC, suggesting that miR-29 occupies a central role in responding to the antagonistic actions of HGF and TGF-β in regulating collagen synthesis in activated and transdifferentiated HSC.

### Overexpression of miR-29 can overcome TGF-β mediated induction of col1A1

**Figure 9:**
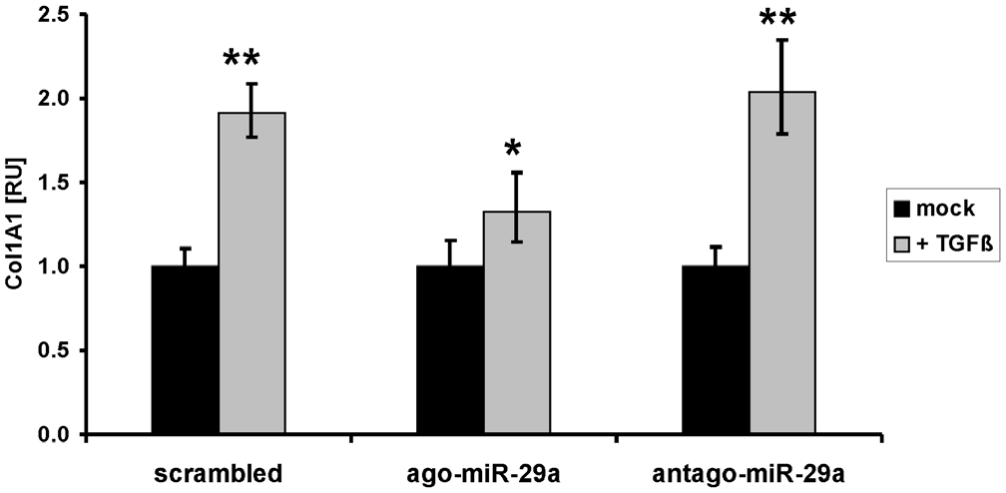
Diminished effect of TGF-β on col1A1 expression in miR-29 treated HSC. The myofibroblastic HSC-T6 cells were transfected with scrambled miRNA, miR-29 mimic (ago-miR-29), or with a miR-29 silencer (antago-miR-29). TGF-β treatment resulted in highly increased col1A1 expression in HSC cells treated with scrambled miRNA or with antago-miR-29, but only a moderate col1A1 induction in miR-29 overexpressing HSC after transfection with ago-miR-29.

After demonstrating that repression of the collagen-inhibiting miR-29 is an important downstream TGF–β effect, we studied if miR-29 overexpression can overcome the profibrogenic features mediated by TGF-β such as col1A1 induction. For this purpose, HSC-T6 cells were transfected with miR-29a mimics (ago-miR29a) in comparison to scrambled or miR-29-silencing miRNA (antago-miR-29). Transfection of HSC with miR-29a mimics lead to a 10-fold overexpression of miR-29a. In HSC that express low levels of miR-29a due to transfection with scrambled miRNA or with a miR-29a silencer, TGF-β treatment highly induced col1A1 synthesis. However, col1A1 induction by TGF-β was only marginal in HSC, that highly overexpress miR-29a after transfection with miR-29a mimics (Figure 9). Thus, miR-29a is able to lower significantly the profibrogenic features of TGF-β in collagen induction.

## Discussion

In the present study we investigated the effects of the opposing action of TGF-β and HGF on miR-29 regulated collagen synthesis by HSC during liver fibrosis. After transdifferentiation into myofibroblasts, HSC constitute the main matrix producing cell type of the fibrotic liver [1], [4]. Our findings demonstrate that myofibroblastic transdifferentiation is accompanied by the loss of HGF synthesis on the one hand, and a tremendous increase of Met receptor expression on the other hand (Figure 1). Previous data have shown that the transition of HSC into myofibroblasts is triggered by paracrine but also by autocrine TGF-β stimulation [12], [51]. Accordingly, TGF-β treatment of HSC leads to decreased HGF expression [29], but enhanced Met receptor synthesis (Figure 1). Although Ikeda et al have suggested that Met receptor expression only occurs in myofibroblastic HSC, its upregulation in response to TGF-β and its association with the pro-fibrotic effects of TGF-β has not been previously shown. TGF-β is the main fibrotic mediator that I) induces the synthesis of ECM components such as collagen type-I and -IV and other collagens [19] II) influences the balance of matrix metalloproteinase (MMP) secretion and their inhibitors [52], and III) affects a wide range of growth factor expression profiles in HSC [1], [4]. The opposed effect of TGF-β on HGF expression and its receptor Met demonstrates that the changing role of HSC in the paracrine HGF stimulation - from a HGF-releasing cell type to a HGF-influenced cell type - is embedded in the regenerative and fibrotic alterations upon chronic liver disease.

Thus, HSC take center stage during fibrosis in responding to the opposing effects of TGF-β and HGF on collagen synthesis. In renal fibroblasts, Yang et al. showed that converse signaling of HGF and TGF-β is due to competition for ERK1/2 mediated inhibition of smad2/3 phoshorylation [48]. Upon TGF-β stimulation of HSC smad2/3 act as the main signal transducers initiating ECM synthesis and myofibroblastic α-smooth-muscle actin expression [12], [51], whereas HGF attenuates TGF-β induced Smad-2/3 nuclear translocation and accumulation [48]. However, blocking of the smad pathway by RNA interference displays that not only the Erk1/2 inhibitory effect on the smad2/3 pathway, but also the HGF mediated activation of Akt pathway is involved in repressing the profibrogenic signalling TGF- β by HGF [49].

In the present study, we now collect evidence that in response to the counteracting HGF / TGF-β signals the miR-29 levels in HSC are contrarily regulated. Recent reports have shown that altered miRNA levels are associated with the phenotypical changes of HSC during the myofibroblastic transition process including the induction of ECM proteins [53], [54]. Our *in silico* target analyses identified various miRNA species putatively suppressing collagen synthesis. In this respect, the members of the miR-29 family are the most promising candidates because they are repressed during myofibroblastic transition and they hold highly conserved binding sites in the 3′-UTR of the various subunits of collagen 1 and 4 (Table 1). Indeed, our *in vitro* data reveal a definite inhibition of collagen type IV, that is the most upregulated collagen form in the fibrotic liver [6], by miR-29. These findings are in agreement with the data of Du et al. [33] and recent reports showing the miR-29 regulation of elastin, fibullin and collagen I synthesis [30], [31], [32], [34], [35].

Keeping with the antifibrotic function of miR-29, miR-29 is reduced in liver biopsies after liver intoxication in mice and after chronic liver disease in humans [35]. The reduced levels of miR-29 during fibrosis are associated with an increase of extracellular miR-29 in serum depending on the fibrotic stage (manuscript in preparation). Furthermore, our *in vitro* and *in vivo* studies on HSC or on BDO-treated fibrotic livers, respectively, suggest that the loss of miR-29 in HSC after TGF-β exposure and during liver fibrogenesis leads to the abolishment of collagen type I and IV repression. Conversely, upregulation of miR-29 levels was observed after stimulation of HSC with the antifibrotic mediator HGF (Figure 8), previously shown to inhibit expression of various collagens [21], [48], [49]. Interestingly, our findings proved that upregulation of miR-29a efficiently can overcome the profibrogenic influence of TGF-β on collagen synthesis (Figure 9). Thus, our findings convincingly demonstrate that HGF mediates antifibrotic signals by influencing miR-29 expression and thereby counteracting the profibrotic activity of TGF-β.

## Supporting Information

Figure S1 **α-SMA expression in HSC.** During myofibroblastic transition of primary HSC in culture (A) and in the HSC-T6 cells after HGF (B) and TGF-β treatment (C). SMA expression was shown by immunochemistry using the monoclonal FITC-labeled 1A4 SMA antibody (Sigma Aldrich) (A) or by real-time PCR (B-D). Transfection of HSC-T6 with ago-miR-29a or ago-miR-29b did not result in altered SMA expression when compared to scrambled miRNA treated HSC-T6 cells.
(TIF)

Figure S2 **miR-29 expression in human skin fibroblasts after stimulation with HGF.** Human primary skin fibroblasts, isolated and cultured according to Zhang et al. (J. Cell Sci, 119 (Pt 9): 1886–1895), were not stimulated (/) or treated for 24 h with 40 ng hHGF or 10 ng TGF-β as described in “Material and Methods” for the stimulation procedure of HSC-T6 cells. miR-29a levels were determined by Real Time-PCR analysis.
(TIF)

Table S1 **Oligonucleotides used for PCR assays.**
(DOC)

Table S2 **Oligonucleotides used for dimerization and insertion into the reporter plasmid psiCHECK^TM^-2.**
(DOC)

Table S3 Ranking list of putative collagen targets from miR-29*.
(DOC)

Table S4 **Putative binding sites of the members of the miR-29 family to the 3′-UTR of different collagens.**
(DOC)
