# Supplementary material for: Hepatocyte Growth Factor (HGF) Inhibits Collagen I and IV Synthesis in Hepatic Stellate Cells by miRNA-29 Induction
Source: PLoS One. 2011 Sep 9;6(9):e24568. doi: 10.1371/journal.pone.0024568 (PMC3170366; doi:10.1371/journal.pone.0024568)
Supplement: Table S1 — Oligonucleotides used for PCR assays. (DOC) [file pone.0024568.s003.doc]

**Supplemental Table S1: Oligonucleotides used for PCR assays**

| **A:** Primers used for amplification of the 3´UTR of col4A1 and col4A5 | |
| --- | --- |
|  |  |
| **Primer** | **Sequence** |
| col4A1 F | ATG AGA AGA ACA TAG TGA TGC CC |
| col4A1 R | TAA GAT ACA CAG CAA AGC TTA CAG G |
| col4A5 F | TTC ACT CAC TCT CCC CAA CTT |
| col4A5 R | GAG AAT AAT TCT TAC ATG GGA AGC |
|  |  |
| **B:** Primers used for transcript quantification by Real Time PCR   | **Primer** | **Sequence** | | --- | --- | | HPRT-Rat-F | GAC CGG TTC TGT CAT GTC G | | HPRT-Rat-R | ACC TGG TTC ATC ATC ACT AAT CAC | | col1A1-Rat-F | CAT GTT CAG CTT TGT GGA CCT | | col1A1-Rat-R | GCA GCT GAC TTC AGG GAT GT | | col1A2-Rat-F | CTG CTC AGT ATT CTG ACA AAG GAG | | col1A2-Rat-R | CTC CGA CAG CAC CAG GAG | | col4A1-Rat-F | GCG AAG GGT GAT TGT GGT | | col4A1-Rat-R | AAC CCT GGA AAG CCT CTC TC | | col4A5-Rat-F | GAC CTC GGG GAC AAA AGG | | col4A5-Rat-R | CCA GGA GGA CCT CTG ATT CC | | HGF-Rat-F | CTT CTG CCG GTC CTG TTG | | HGF-Rat-R | TCT TCT CTT CTT CTG TCC TTC TGC | | TGF-ß-Rat-F | CCT GGA AAG GGC TCA ACA C | | TGF-ß-Rat-R | CAG TTC TTC TCT GTG GAG CTG A | | c-met-Rat-F | CAA GAT TGT CAA CAA AAA CAA CG | | c-met-Rat-R | AGC CGA TTG AAT TTC TCA GC | | SMA-F | TGC CAT GTA TGT GGC TAT TCA | | SMA-R | ACC AGT TGT ACG TCC AGA AGC | | pri-miR-29a/b-F | AGC TAT CCA CAA CAC CGA GGT AAG | | pri-miR-29a/b-R | TAT CTA AGA CAG TCA GGC CAC CAG | | pri-miR-29b/c-F | TAC TGC CTG CCT GTA CCT GGA G | | pri-miR-29b/c-R | AGG TCT GAA TCT CAA GGC AGG TG | | |
