# Supplementary material for: Hepatocyte Growth Factor (HGF) Inhibits Collagen I and IV Synthesis in Hepatic Stellate Cells by miRNA-29 Induction
Source: PLoS One. 2011 Sep 9;6(9):e24568. doi: 10.1371/journal.pone.0024568 (PMC3170366; doi:10.1371/journal.pone.0024568)
Supplement: Table S2 — Oligonucleotides used for dimerization and insertion into the reporter plasmid psiCHECKTM-2. (DOC) [file pone.0024568.s004.doc]

**Supplemental Table S2: Oligonucleotides used for dimerization and insertion into the reporter plasmid psiCHECK^TM^-2**

| **Name** | **Sequence of Oligonucleotides** |
| --- | --- |
| miR29a S | TCG AGA ACC GAT TTC AGA TGG TGC TAA GAT CTA ACC  GAT TTC AGA TGG TGC TAG C |
| miR29a A | GGC CGC TAG CAC CAT CTG AAA TCG GTT AGA TCT TAG  CAC CAT CTG AAA TCG GTT C |
| col1A1 S | TCG AGT GGG AAG GAA TTT CTG GTG CTA TAG AAT CTG C |
| col1A1 A | GGC CGC AGA TTC TAT AGC ACC AGA AAT TCC TTC CCA C |
| col1A1 mut S | TCG AGT GGG AAG GAA TTT CTG TCG CTA TAG AAT CTG C |
| col1A1 mut A | GGC CGC AGA TTC TAT AGC GAC AGA AAT TCC TTC CCA C |
| col1A2 S | TCG AGC GAC ACA AAG GTG CTA ATT AGT AGG C |
| col1A2 A | GGC CGC CTA CTA ATT AGC ACC TTT GTG TCG C |
| col1A2 mut S | TCG AGC GAC ACA AAG TTG ATA CTT AGT AGG C |
| col1A2 mut A | GGC CGC CTA CTA AGT ATC AAC TTT GTG TCG C |
| col4A1 S | TCG AGG CCG CCG TCA CAA CAT GGT GCT ACG C |
| col4A1 A | GGC CGC GTA GCA CCA TGT TGT GAC GGC GGC C |
| col4A1 mut S | TCG AGG CCG CCG TCA CAA CAT AGT GCG ACG C |
| col4A1 mut A | GGC CGC GTC GCA CTA TGT TGT GAC GGC GGC C |
| col4A5 S | TCG AGA AGT ATT CTT TTT CAT GGT GCT ACG C |
| col4A5 A | GGC CGC GTA GCA CCA TGA AAA AGA ATA CTT C |
| col4A5 mut S | TCG AGA AGT ATT CTT TTT CAT AGT GCG ACG C |
| col4A5 mut A | GGC CGC GTC GCA CTA TGA AAA AGA ATA CTT C |

S: sense oligonucleotide strand A: antisense oligonucleotide strand
