# Supplementary material for: Hepatocyte Growth Factor (HGF) Inhibits Collagen I and IV Synthesis in Hepatic Stellate Cells by miRNA-29 Induction
Source: PLoS One. 2011 Sep 9;6(9):e24568. doi: 10.1371/journal.pone.0024568 (PMC3170366; doi:10.1371/journal.pone.0024568)
Supplement: Table S3 — Ranking list of putative collagen targets from miR-29*. (DOC) [file pone.0024568.s005.doc]

**Supplemental Table S3: Ranking list of putative collagen targets from miR-29***

| **Putative Targets** | **High Conserved Sites** | **Poorly Conserved Sites** | **Total context score*** |
| --- | --- | --- | --- |
| **col4A5** | **2** | **1** | **-1.03** |
| **col5A2** | **4** | **0** | **-0.7** |
| **col4A3** | **2** | **1** | **-0.68** |
| **col9A1** | **1** | **1** | **-0.67** |
| **col4A4** | **2** | **0** | **-0.65** |
| **col11A1** | **2** | **0** | **-0.61** |
| **col5A3** | **3** | **0** | **-0.6** |
| **col1A1** | **3** | **0** | **-0.58** |
| **col7A1** | **2** | **0** | **-0.56** |
| **col3A1** | **1** | **0** | **-0.5** |
| **col4A1** | **2** | **0** | **-0.44** |
| **col2A1** | **1** | **0** | **-0.43** |
| **col15A1** | **1** | **0** | **-0.4** |
| **col6A3** | **1** | **0** | **-0.39** |
| **col5A2** | **1** | **0** | **-0.39** |
| **col22A1** | **1** | **1** | **-0.24** |
| **col4A2** | **1** | **0** | **-0.23** |
| **col19A1** | **1** | **0** | **-0.21** |
| **col1A2** | **1** | **0** | **-0.18** |
| **col24A1** | **1** | **0** | **-0.16** |
| **col16A1** | **1** | **0** | **-0.09** |

* identified by Targetscan [42].
