# Supplementary material for: Hepatocyte Growth Factor (HGF) Inhibits Collagen I and IV Synthesis in Hepatic Stellate Cells by miRNA-29 Induction
Source: PLoS One. 2011 Sep 9;6(9):e24568. doi: 10.1371/journal.pone.0024568 (PMC3170366; doi:10.1371/journal.pone.0024568)
Supplement: Table S4 — Putative binding sites of the members of the miR-29 family to the 3′-UTR of different collagens. (DOC) [file pone.0024568.s006.doc]

**Supplemental Table S4 : Putative binding sites of the members of the miR-29 family to the 3´-UTR of different collagens.**

|  | **COL4A1** | **predicted consequential pairing of target region (top) and miRNA (bottom)** | **seed match** |
| --- | --- | --- | --- |
| **Position 29-35*** | rno-miR-29a | 5' ...GCCGCCGUCACAACAUGGUGCUA...                       |||||||   3'     AUUGGCUAAAGUCUACCACGAU | 8mer |
|  | rno-miR-29b | 5'  ...GCCGCCGUCACAACAUGGUGCUA...                        \|\|\|\|\|\|\|   3'     UUGUGACUAAAGUUUACCACGAU | 8mer |
|  | rno-miR-29c | 5' ...GCCGCCGUCACAACAUGGUGCUA...                       \|\|\|\|\|\|\|   3'     AUUGGCUAAAGUUUACCACGAU | 8mer |
| **Position 332-338** | rno-miR-29a | 5' ...CACAAGAAACCCAAAGGUGCUAG...                       ||||||    3'    AUUGGCUAAAGUCUACCACGAU | 7mer-1A |
|  | rno-miR-29b | 5'  ...CACAAGAAACCCAAAGGUGCUAG...                        \|\|\|\|\|\|    3'    UUGUGACUAAAGUUUACCACGAU | 7mer-1A |
|  | rno-miR-29c | 5'  ...CACAAGAAACCCAAAGGUGCUAG...                        \|\|\|\|\|\|    3'     AUUGGCUAAAGUUUACCACGAU | 7mer-1A |

|  | **COL4A5** | **predicted consequential pairing of target region (top) and miRNA (bottom)** | **seed match** |
| --- | --- | --- | --- |
| **Position 134-140** | rno-miR-29a | 5'  ...CACUGCUACCACCAAUGGUGCUA...                        |||||||   3'      AUUGGCUAAAGUCUACCACGAU | 8mer |
|  | rno-miR-29b | 5'   ...CACUGCUACCACCAAUGGUGCUA...                         \|\|\|\|\|\|\|   3'      UUGUGACUAAAGUUUACCACGAU | 8mer |
|  | rno-miR-29c | 5'  ...CACUGCUACCACCAAUGGUGCUA...                        \|\|\|\|\|\|\|   3'      AUUGGCUAAAGUUUACCACGAU | 8mer |
| **Position 404-410*** | rno-miR-29a | 5' ...AAGUAUUCUUUUUCA--UGGUGCUA...                  |||||  |||||||   3'       AUUGGCUAAAGUCUACCACGAU | 8mer |
|  | rno-miR-29b | 5' ...AAGUAUUCUUUUUCA--UGGUGCUA...                  \|\|\|\|\|  \|\|\|\|\|\|\|   3'      UUGUGACUAAAGUUUACCACGAU | 8mer |
|  | rno-miR-29c | 5' ...AAGUAUUCUUUUUCA--UGGUGCUA...                  \|\|\|\|\|  \|\|\|\|\|\|\|   3'       AUUGGCUAAAGUUUACCACGAU | 8mer |

**Yellow shadowed: binding sites in the 3´- ÚTR of collagen subtypes , that are chosen for further studies*

|  | **COL1A1** | **predicted consequential pairing of target region (top) and miRNA (bottom)** | **seed match** |
| --- | --- | --- | --- |
| **Position 861-867** | rno-miR-29a | 5'  ...CAGUUUGGUAUCAAAGGUGCUAC...                        ||||||    3'     AUUGGCUAAAGUCUACCACGAU | 8mer |
|  | rno-miR-29b | 5' ...CAGUUUGGUAUCAAA-GGUGCUAC...                  \|\|\|\|\| \|\|\|\|\|\|    3'    UUGUGACUAAAGUUUACCACGAU | 8mer |
|  | rno-miR-29c | 5' ...CAGUUUGGUAUCAAA-GGUGCUAC...                  \|\|\|\|\| \|\|\|\|\|\|    3'     AUUGGCUAAAGUUUACCACGAU | 8mer |
| **Position 903-909*** | rno-miR-29a | 5' ...GUGGGAAGGAAUUUC---UGGUGCUA...                  |||||   |||||||   3'        AUUGGCUAAAGUCUACCACGAU | 8mer |
|  | rno-miR-29b | 5' ...GUGGGAAGGAAUUUC---UGGUGCUA...                  \|\|\|\|\|   \|\|\|\|\|\|\|   3'       UUGUGACUAAAGUUUACCACGAU | 8mer |
|  | rno-miR-29c | 5' ...GUGGGAAGGAAUUUC---UGGUGCUA...                  \|\|\|\|\|   \|\|\|\|\|\|\|   3'        AUUGGCUAAAGUUUACCACGAU | 8mer |
| **Position 1021-1027** | rno-miR-29a | 5'  ...UUUUCUUUUCCUGACGGUGCUAU...               ||||     ||||||    3'    AUUGGCUAAAGUCUA-CCACGAU | 7mer-1A |
|  | rno-miR-29b | 5'   ...UUUUCUUUUCCUGACGGUGCUAU...                \|\|\|\|     \|\|\|\|\|\|    3'    UUGUGACUAAAGUUUA-CCACGAU | 7mer-1A |
|  | rno-miR-29c | 5'  ...UUUUCUUUUCCUGACGGUGCUAU...               \|\|\|\|     \|\|\|\|\|\|    3'    AUUGGCUAAAGUUUA-CCACGAU | 7mer-1A |

|  | **COL1A2** | **predicted consequential pairing of target region (top) and miRNA (bottom)** | **seed match** |
| --- | --- | --- | --- |
| **Position 506-512*** | rno-miR-29a | 5'     ...UGCUUUCGACACAAAGGUGCUAA...                           ||||||    3'        AUUGGCUAAAGUCUACCACGAU | 7mer-1A |
|  | rno-miR-29b | 5'      ...UGCUUUCGACACAAAGGUGCUAA...                            \|\|\|\|\|\|    3'        UUGUGACUAAAGUUUACCACGAU | 7mer-1A |
|  | rno-miR-29c | 5'     ...UGCUUUCGACACAAAGGUGCUAA...                           \|\|\|\|\|\|    3'        AUUGGCUAAAGUUUACCACGAU | 7mer-1A |

**Yellow shadowed: binding sites in the 3´-ÚTR of collagen subtypes , that are chosen for further studies*
